# Urban health, the right to the city, and the communities of Manguinhos in Rio de Janeiro

**DOI:** 10.1590/S0104-59702023000100063

**Published:** 2023-11-10

**Authors:** Luís Carlos Soares Madeira Domingues, Rosângela Lunardelli Cavallazzi

**Affiliations:** i Tecnologista em Saúde Pública, Fundação Oswaldo Cruz;; doutorando,Programa de Pós-graduação em Urbanismo/Universidade Federal do Rio de Janeiro. luis.madeira@fiocruz.br; ii Professora e pesquisadora, Programa de Pós-graduação em Urbanismo/Universidade Federal do Rio de Janeiro;; Programa de Pós-graduação em Direito/Pontifícia Universidade Católica do Rio de Janeiro. rosangela.cavallazzi@gmail.com

**Keywords:** Urban health, Social commitment to health, Right to the city, Housing needs, Communities of Manguinhos, Saúde urbana, Determinação social da saúde, Direito à cidade, Necessidades habitacionais, Comunidades de Manguinhos

## Abstract

This article proposes a critical reading of the urbanistic and social historical context of the communities of Manguinhos, considering the field of urban health as a theoretical and methodological foundation based on the paradigm of the social commitment to health and the right to the city. Our demarcation of this analysis to identify the critical processes in the social commitment to health considered the relevance of overcoming housing needs as an indispensable condition for the right to the city and to health. The fact that these processes persist, even after the vast investments made in Manguinhos, indicates the need to review related public policies and their effective implementation to improve conditions for this population.

## Resumo

O artigo propõe uma leitura crítica do contexto histórico urbanístico e social das comunidades de Manguinhos considerando a fundamentação teórico-metodológica do campo da saúde urbana, apoiada no paradigma da determinação social da saúde e do direito à cidade. O recorte de análise para a identificação dos processos críticos de determinação social da saúde considerou a relevância da superação das necessidades habitacionais como condição indispensável para o direito à cidade e à saúde. A constatação da permanência dos processos mencionados, mesmo após os vultosos investimentos em urbanização realizados em Manguinhos, aponta para a necessidade de revisão das políticas públicas relacionadas e a inserção efetiva e emancipatória da população nessas políticas.

As cidades brasileiras refletem espacialmente a desigualdade socioeconômica e os graves descompassos históricos e estruturais da nossa sociedade, mas também produzem essas desigualdades e interferem em suas determinações. Após alguns avanços no campo legal e na formulação de políticas públicas que buscavam atuar nos mecanismos de reprodução desse quadro, a partir da Constituição cidadã de 1988, nos encontramos em um contexto de retrocessos nas políticas públicas e na concepção e no acesso aos direitos sociais, que se consolida, a partir de 2016, com o afastamento da presidenta Dilma Rousseff, e a chegada ao poder de uma coalizão conservadora no governo federal no Brasil. Esse contexto tem influência no avanço da intolerância e violência política, no esvaziamento do processo democrático e da participação social nas políticas públicas, na restrição à transparência e ao monitoramento das informações públicas, mas também reflete a política de austeridade e o consequente abandono de políticas redistributivas (Santos Junior, Leite, Muller, 2020).

Esses problemas já representavam desafios históricos na condução de políticas públicas, incluídas aquelas que pretendiam atuar focalmente para reversão de desigualdades socioespaciais como as políticas de urbanização de favelas. Mas o impacto desse novo contexto sobre as cidades e principalmente sobre as populações mais vulnerabilizadas é dramático, e seu agravamento fica mais evidente após o início da pandemia da covid-19 não somente em indicadores de morbidade e mortalidade, mas também em indicadores sociais, como na queda da renda e do emprego, no aumento acentuado da fome e da insegurança alimentar e na multiplicação de famílias dormindo nas ruas (Rede Penssan, 2022; Ahmed et al., jan. 2022). Nas Américas, em meados de 2021, se verificavam os piores indicadores internacionais de contaminação e mortes entre as populações dos países, o que sugere a má gestão pública da pandemia, o negacionismo científico de vários desses governos e sua associação, especialmente, na América Latina, com as desigualdades sociais e a discriminação contra as classes de menor renda (Agostoni, Ramacciotti, Lopes, 2022, p.564). Os efeitos da pandemia sobre as populações vulneráveis, com a ampliação da exposição e a vulnerabilidade das famílias residentes em favelas e periferias, explicitaram a importância das condições urbanas e habitacionais para o enfrentamento da crise sanitária e a preservação da vida, especialmente na relação entre desigualdades socioeconômicas, a estrutura socioespacial das cidades e as iniquidades em saúde (ONU Mujeres, 2020).

Um dos desafios que se impõem diante da atual crise social, política, ambiental e sanitária é a realização de uma reflexão que articule o enfrentamento das situações emergenciais no apoio às famílias em territórios vulneráveis com propostas que incorporem estratégias de médio e longo prazos, visando transformar os processos de determinação social que afetam a saúde e a qualidade de vida dessa população, bem como as condições de enfrentamento dessa e de futuras emergências sanitárias e climáticas. É importante ressaltar que pudemos acompanhar experiências exitosas sobre as ações emergenciais em territórios vulnerabilizados, como a constituição de estratégias de vigilância epidemiológica, de mobilização social e de redes intersetoriais e solidárias, que mostram a capacidade de articulação entre sociedade civil e movimentos organizados nos e dos territórios, em contraponto à ineficácia de ação do poder público na definição de estratégias de proteção e cuidado com a população (Ribeiro, 2020). A percepção sobre a necessidade de medidas estruturais e transformadoras das iniquidades em saúde observadas na cidade aponta para a oportuna recuperação da experiência acumulada pela sociedade em políticas públicas que lidam com o enfrentamento do *deficit*[1] habitacional e a inadequação[2] de domicílios duráveis, ampliando o acesso à terra e à moradia digna.[3] Iniciativas com esse objetivo, entretanto, enfrentam dificuldades em se constituir como prioridade por meio de políticas efetivas, principalmente com a aproximação do fim da emergência sanitária.

O objetivo deste trabalho é contribuir com essa reflexão a partir do contexto histórico urbanístico e social das comunidades de Manguinhos, considerando a fundamentação teórico-metodológica do campo da saúde urbana, apoiada no paradigma da determinação social da saúde e do direito à cidade, buscando identificar os processos críticos de determinação da saúde e sua permanência no tempo, com um olhar específico sobre os processos que ajudam a compreender a constituição e as características do ambiente construído e sua influência na saúde da população. Para tanto, é proposto um diálogo com a abordagem de Breilh (2010) e Santos (2006), em que se assume que a produção e reprodução do espaço urbano, influenciadas por ações e políticas públicas, definem condicionantes urbanísticas que podem ser compreendidas como características do ambiente construído e dinâmicas urbanas, que se verificam no nível local e na própria estrutura da cidade, e constituiriam processos estruturais de determinação social da saúde. Essas condicionantes, por outro lado, também reforçam as iniquidades em saúde, por exemplo, quando as características do ambiente construído impõem restrições à mobilidade urbana e ao acesso a serviços e equipamentos públicos, bem como ao acentuar riscos ambientais e à saúde, diante de sua localização relativa e da precariedade das condições habitacionais e de saneamento (Kowarick, 2020; Maricato, 2013).

Essas condicionantes apresentam também forte influência na percepção simbólica da sociedade sobre as favelas, construção histórica pautada na estigmatização e associação desses assentamentos à precariedade e à criminalização (Silva, Menezes, 2019), que acabam por legitimar, junto à sociedade, o desenho e a forma como as políticas públicas são implementadas em territórios vulnerabilizados. Assim, compreende-se que a transformação das condições de vida dessa população não passa somente pela reestruturação e requalificação do ambiente construído, mas pelo respeito à história de vida das comunidades, por meio da recuperação e valorização da memória e identidade da população com o seu território, em um processo de ressignificação desses territórios como potência (Barbosa, Silva, 2013).

O trabalho utiliza, como caso referência, os assentamentos precários[4] do Complexo[5] de Manguinhos que foram objeto do Plano de Aceleração do Crescimento Urbanização de Assentamentos Precários, também identificado como PAC Manguinhos, considerando que essa constitui mais uma entre as iniciativas de políticas públicas que preveem intervenção sobre o ambiente construído e social em assentamentos precários que, apesar de apresentar potencial para contribuir com a reversão das iniquidades em saúde, tradicionalmente, na experiência internacional e nacional, não são planejadas, implementadas e avaliadas considerando os processos críticos de determinação da saúde, implicando a permanência desses processos e, portanto, dos riscos à vida e à saúde dessa população (Corburn, Sverdlik, 24 mar. 2017). Nessas também, raramente, se considera ou se avalia o impacto das decisões projetuais e de sua implementação na saúde e na qualidade de vida da população que, geralmente, não é incluída nos processos decisórios e no controle social das ações realizadas em seu contexto de vida, e, mesmo quando incluída, esses processos não garantem sua participação e influência efetiva.

Este trabalho reconhece a importância da análise da organização social e dos processos participativos experimentados pela população para a transformação da realidade social em uma perspectiva emancipatória, diante da histórica lógica populista e clientelista na relação entre populações periféricas e o poder público. Essa lógica, baseada em troca de favores e em não incluir, de forma efetiva, a população nos processos decisórios, impõe limites aos movimentos associativos de bairro e iniciativas coletivas que estejam compromissadas com uma perspectiva promotora de saúde para os territórios. Ao criar restrições à participação efetiva da população em planejamento, implementação, monitoramento e avaliação de ações e políticas públicas ocorre o comprometimento dos resultados da ação pública não somente sobre o território, como sobre possibilidades concretas de acesso aos direitos sociais e ao direito à cidade.

Apresenta-se, inicialmente, breve síntese com a fundamentação teórica que articula o campo da saúde urbana e o direito à cidade, seguida de alguns apontamentos sobre o contexto histórico urbanístico e social de Manguinhos que sejam relevantes para a identificação dos processos de produção e reprodução urbanas que determinaram sua realidade física e social e a reflexão sobre a influência desses processos e das características do ambiente construído sobre a saúde. Verifica-se, posteriormente, a permanência desses processos e as características mencionadas, tendo em vista as transformações realizadas no espaço urbano pelo PAC Manguinhos, especialmente os impactos sobre o acesso à moradia digna e a superação das inadequações habitacionais. Por fim, traçam-se algumas considerações abordando a oportunidade oferecida por políticas que têm o potencial de atuar sobre o *deficit* habitacional e a inadequação fundiária, da moradia e de infraestrutura, considerando sua importância para a qualidade de vida e saúde da população, assim como para o enfrentamento de emergências sanitárias e eventos climáticos, e a necessidade de se refletir sobre novos desenhos para essas políticas, seja em seus princípios e estratégias de atuação sobre o ambiente construído, seja em seus instrumentos, instâncias e processos de gestão democrática.

## Saúde urbana em uma abordagem crítica e o direito à cidade

A conexão entre saúde e planejamento urbano passa a existir, implícita ou explicitamente, desde quando se verifica a mudança das comunidades humanas para uma vida duradoura e em assentamentos mais densos, com relevante impacto sobre os padrões de morbidade, mortalidade e etiologia (De Leeuw, 2017, p.5). Há um longo histórico a respeito da articulação entre esses dois campos de conhecimento, mas é a partir do século XVIII que Foucault (2016, p.411) identifica o estabelecimento de uma medicina coletiva, social, urbana, associada à necessidade de organizar o corpo urbano de maneira coerente e homogênea, e a regê-lo por um poder único e bem regulamentado, como instância de controle social. O crescimento das tensões políticas e o surgimento ou ampliação de um sentimento de medo, angústia, diante da inquietude político-sanitária no interior das cidades eram justificativas utilizadas para a adoção de modelos de organização médica, no Ocidente, baseados na exclusão ou na vigilância e no controle do estado de saúde da população, mediante uma análise minuciosa da cidade, com registro permanente, frequentes intervenções autoritárias, constituindo a higiene pública como o controle político-científico desse meio, tendo forte influência no urbanismo em sua origem, como campo científico e em sua relação com a saúde pública.

Entre os diversos paradigmas explicativos para os problemas de saúde, até meados do século XIX predominava a teoria dos miasmas, e muitas intervenções de saúde no meio urbano focalizaram sua remoção física – lixo, águas residuais, poluição do ar e similares – e das pessoas consideradas indesejáveis e doentes, quando, nas últimas décadas do século XIX, se afirmou um novo paradigma para a explicação do processo saúde-doença com o trabalho de bacteriologistas como Koch e Pasteur (Buss, Pellegrini Filho, 2007, p.79). A perspectiva de que problemas de saúde pudessem ser tratados com a eliminação de um único organismo teve forte impacto na atuação sobre uma infinidade de conceitos abstratos e inter-relacionados da realidade urbana. A partir de então observa-se uma permanente tensão entre a abordagem baseada na microbiologia e nos sucessos da teoria dos germes e outra, centrada no estudo da influência das condições sociais, econômicas e ambientais na saúde dos indivíduos, conformando, ao longo do século XX, um afastamento gradativo entre os campos da saúde pública e do urbanismo, cujas práticas continuam a produzir desigualdades sociais e iniquidades em saúde (p.80).

Esses campos voltam a se reaproximar nestes últimos 50 anos, quando se inicia uma nova perspectiva no sentido de considerar que as condições sociais influenciam decisivamente a saúde e ressaltar a importância da intersetorialidade para a promoção da saúde e do bem-estar da população (Hancock, Duhl, 1988), com destaque à Declaração de Alma-Ata, adotada em 1978, com conteúdo enfatizado na Carta de Ottawa, em 1986. No entanto, para De Leeuw (2017), as propostas que consideram a saúde na agenda de políticas públicas urbanas e nas reflexões e técnicas de planejamento e projeto urbano permaneceram marginais em face das abordagens cartesianas e reducionistas observadas na relação entre os campos do urbanismo e da saúde pública, que acabam por reproduzir as relações de poder na sociedade e um modelo físico-estrutural no qual o ambiente construído é concebido, fundamentalmente, em seu aspecto formal e estético.

A literatura de saúde urbana surge, nesse contexto, com foco na análise dos efeitos da urbanização e dos atributos físicos da cidade e seus bairros e/ou vizinhanças sobre a saúde humana, e com o intuito de definir estratégias de enfrentamento dos desafios gerados por elas (Frumkin, Frank, Jackson, 2004). O conceito de saúde urbana considera que a urbanização não produz somente efeitos benéficos, conhecidos como as vantagens urbanas, mas pode acarretar danos sociais, econômicos e ambientais de grande impacto, difíceis de mensurar de forma precisa (Opas, 2007, p.49). Se considerarmos o papel do ambiente físico e social em condicionar a saúde das pessoas, levando em conta as características da cidade e seu entorno, e do contexto em que as pessoas vivem, podemos nos aproximar da compreensão do processo saúde-doença e da cidade/urbano como exposição, modulando de forma benéfica ou danosa a saúde de suas populações residentes (Vlahov et al., 2007; Caiaffa et al., 2008), e, observando as evidências na relação entre condições urbanas, os processos de urbanização e a saúde, seria possível relacionar a pobreza urbana e a concentração de parte relevante da carga global da doença (WHO, 2016).

O debate sobre os determinantes e a determinação social da saúde se faz relevante em torno da distinção e clareza de seus conteúdos e implicações. A determinação social da saúde é uma das categorias centrais da proposta da epidemiologia crítica que se constitui sobre a crítica à abordagem dos determinantes sociais. A reflexão sobre as diferentes concepções da determinação social da saúde se relaciona com a distinção do saber, a construção histórica e os projetos da saúde coletiva no Brasil e da medicina social na América Latina, anunciados a partir de meados da década de 1970 e, posteriormente, desenvolvidos e complementados por muitos autores, especialmente dessa corrente latino-americana, no que veio a se configurar como um rompimento epistemológico e de práxis em relação à saúde pública (Borghi, Oliveira, Sevalho, 2018, p.873).

A determinação social da saúde na perspectiva da saúde urbana propõe uma construção inovadora do espaço urbano da saúde, a partir das contribuições da abordagem crítica de campos disciplinares como a geografia, a ecologia urbana, o direito urbanístico e o urbanismo articulados à epidemiologia crítica que, embora intimamente relacionadas, possuem suas especificidades. Esse esforço parte da crítica ao modelo de produção e reprodução urbanas que reforça a concentração de renda nas cidades, exclui e segrega grupos sociais, em um contexto acelerado de acumulação econômica, que consiste em um sistema incompatível com a reprodução humana de forma digna, próprio da maior parcela da população urbana. Ao lado disso, esse modelo e essa dinâmica produzem impactos ambientais em diversas escalas, em especial diante de sua demanda por energia e recursos materiais, impondo um padrão insustentável à vida.

Segundo Breilh (2010, p.84), uma "reflexão emancipatória sobre os problemas de saúde da cidade tem que superar as restrições clássicas da teoria do espaço urbano e as concepções reducionistas de saúde e meio ambiente, para abordar a necessidade coletiva", buscando superar o sentido empírico da noção de "lugar" que reduz o desenho das políticas a uma perspectiva apenas funcionalista entre fatores e o processo saúde-doença. Coerente com essa reflexão, Santos (2006, p.14) considera que ações humanas e objetos do espaço interagem e se determinam constantemente, por meio de um movimento permanente de dissolução e de recriação do sentido, logo, são indissociáveis durante uma análise geográfica. A produção e reprodução do espaço, com sua sucessão interminável de formas-conteúdo que se dão com um crescente conteúdo de ciência, de técnicas e de informação, constituem um elemento central de sua compreensão. A presença da ciência, da técnica e da informação nos processos de remodelação do território seria assim essencial às produções hegemônicas, que necessitam do meio geográfico para sua realização (p.35-36).

Santos (2006) afirma ainda que os processos de mudança social, econômico e político da sociedade resultam na transformação do espaço, e que cada período histórico busca alterar, renovar e adaptar o espaço e a paisagem para atender aos novos paradigmas do modo de produção e reprodução social. Nesse sentido, o espaço seria uma variável fundamental para se compreender a história, e ambos são variáveis importantes para se compreender os processos de determinação da saúde e seu movimento. Para Breilh (2010, p.88-90), a saúde se desenvolve como um processo entre as dimensões espaciais do geral, do particular e do singular, onde as determinações e contradições estruturais mais amplas influenciam os modos de vida coletivos e os estilos de vida individuais. Nestas duas últimas dimensões se forjam as tendências desfavoráveis à saúde e os processos saudáveis e protetores, físicos e psicológicos, cuja influência é condicionada pelas características individuais de cada um, devendo considerar uma certa autonomia das citadas dimensões às demais, o que permite gerar novas condições de saúde a partir das esferas locais.

O reconhecimento do território não somente como delimitação de uma área, mas também dos processos e territorialidades que o conformam, é passo importante para identificar essas determinações sociais e as situações de saúde, bem como para a tomada de decisões (Gondim, Monken, 2017). As estratégias territorializadas de saúde sintonizadas com os princípios da saúde coletiva latino-americana permitem articular as estratégias de promoção da saúde às estratégias de atenção e vigilância em saúde, voltadas para o atendimento de necessidades de saúde da população no sentido da integralidade do cuidado, bem como fortalecer processos de descentralização e democratização da gestão pública.

No Brasil, o Programa Saúde da Família/Estratégia Saúde da Família, também identificado como PSF/ESF, e o Programa de Agentes Comunitários de Saúde, também identificado como Pacs, pertencem ao Sistema Único de Saúde (SUS) e são exemplos dessas estratégias. A intersetorialidade é um princípio central nessa reflexão, pois permite ampliar o leque de ações sociais voltadas para a produção social da saúde nos territórios no enfrentamento de problemas com o olhar atento às especificidades do território, reconhecendo que os processos de determinação não podem ser enfrentados somente no âmbito do sistema de serviços de saúde (Teixeira, Solla, 2006, p.72).

Entre os apontamentos da teoria urbana crítica que contribuem com esse debate, Harvey (jul.-dez. 2012, p.74) observa uma conexão estreita entre o desenvolvimento do capitalismo e a urbanização e entre urbanização e direitos sociais, ao afirmar que "a urbanização sempre foi um fenômeno de classe, já que o excedente é extraído de algum lugar e de alguém, enquanto o controle sobre sua distribuição (investimentos públicos) repousa nas mãos de poucos". Vivemos em um momento em que os direitos de propriedade privada e a taxa de lucro permanecem se sobrepondo a todas as outras noções de direito, e a terra e sua localização nas cidades consistem em um elemento central da disputa capitalista.

O ataque especulativo sobre a terra concebida como um elemento estrutural de acumulação e de materialização dos ganhos financeiros é um dos aspectos da financeirização também estudado por Rolnik (2015, p.26), que avalia igualmente processos, em todo o mundo, de "desconstrução da habitação como um bem social e de sua transmutação em mercadoria e ativo financeiro". A referida autora busca também relacionar a crise global de insegurança da posse ao avanço do complexo imobiliário-financeiro, aos processos de privatização do patrimônio público e de serviços urbanos básicos, ao aprofundamento da desigualdade social e seu impacto sobre direito à moradia, insurgências, conflitos e violência nas cidades.

O avanço da pauta neoliberal sobre a saúde pública converge com esse cenário, o que pode ser observado na crescente privatização do SUS, por exemplo, na terceirização da rede de atenção básica às organizações sociais e no financiamento público da rede de atendimento do setor privado, sem a adequada coordenação, associado ao subfinanciamento crônico do sistema, desde sua criação, em 1988, com impacto negativo sobre os princípios adotados pelo SUS de universalização, integralidade e qualidade dos serviços prestados.

Essas reflexões explicitam a relevância da agenda da reforma urbana e a defesa do direito à cidade para a agenda da saúde urbana e da equidade em saúde, resultado da formação de um pensamento crítico que, desde os anos 1960, define princípios comuns para as cidades e aponta para a defesa da função social da propriedade e da cidade, consolidados na Constituição de 1988 e regulamentados no Estatuto da Cidade, em 2001.

Essa agenda explicita, também, a defesa da equidade e da justiça social e ambiental, com a justa distribuição social e espacial de recursos e dos benefícios e ônus decorrentes da urbanização, a superação da dicotomia, segregação e desmitificação das áreas de favelas e periferias, onde se concentra a pobreza urbana na cidade, bem como da descentralização e municipalização da política urbana e da gestão democrática da cidade.

Ao refletir sobre o direito à cidade, Harvey (jul.-dez. 2012, p.74) afirma que este deve ser compreendido como um direito coletivo, antes de um direito individual, já que essa transformação depende inevitavelmente do exercício de um poder coletivo de moldar o processo de urbanização; portanto, se afasta da liberdade individual de acesso a recursos urbanos e assume a perspectiva de "mudar a nós mesmos pela mudança da cidade". Nesse sentido, esse direito não deve ser entendido como um direito ao que já existe, mas como um direito de reconstruir e recriar a cidade como um corpo político com uma imagem totalmente distinta, compromissado com a erradicação da pobreza e da desigualdade social e com a reversão do processo de degradação ambiental. A construção da cidade é espaço de disputas e base fundamental para os processos de acumulação do capital, portanto, um maior controle democrático sobre a produção e utilização do excedente é estratégico, dado que o processo de desenvolvimento urbano é o seu principal caminho de utilização. Estabelecer uma gestão democrática sobre o planejamento, a gestão, a regulação, o controle e a implementação de políticas públicas e intervenções urbanas constitui, assim, uma das condições para o direito à cidade.

A luta pelo direito à cidade passou a ser depositária das expectativas de mudança, das projeções de sustentabilidade, justiça, democracia e igualdade na cidade e horizonte de emancipação e de articulação de diversos movimentos sociais como os que lutam por moradia digna, acesso à terra e segurança da posse, acesso aos bens e serviços públicos e espaços públicos da cidade com qualidade. A associação pragmática e estratégica entre a agenda urbana e a agenda da saúde coletiva, em diálogo com as pautas de gênero, raça, juventude e valorização da cultura, tem buscado viabilizar a construção de caminhos que ampliem a perspectiva de futuro e de transformação do presente.

## O processo de produção e reprodução do espaço urbano da cidade no contexto de Manguinhos

O histórico e o diagnóstico urbanístico de Manguinhos apresentam o que podemos considerar os efeitos no espaço do processo de produção e reprodução de uma cidade capitalista em um país periférico, diante das especificidades históricas da evolução urbana da cidade do Rio de Janeiro e do suporte oferecido pelo ambiente físico. Esse processo consiste em um dos macroprocessos de determinação social da saúde, como já mencionado, e os elementos constituintes do ambiente construído são por ele determinados, mas se tornam determinantes ao se constituir como mediadores dos processos de determinação da saúde em suas diversas dimensões de análise, ao limitar ou potencializar processos promotores de saúde.

O bairro de Manguinhos localiza-se na zona Norte do município do Rio de Janeiro em uma região que, em sua origem, se encontrava às margens da baía de Guanabara, ocupada por matas e manguezais, e se caracterizava por ser predominantemente plana e cortada pelos rios Faria-Timbó, Jacaré e canal do Cunha que se encontram próximos de sua foz.

A implantação da Estrada de Ferro Leopoldina, na década de 1880-1890, em seguida à passagem pela região da Estrada de Ferro Central do Brasil, inaugurada em 1858, representou o início do aterramento de diversas regiões do subúrbio e da baixada Fluminense, entre elas os manguezais do estuário de Manguinhos, e assumiu, nesse primeiro momento, o papel de principal indutor de expansão de ocupação e de perspectivas de desenvolvimento para a região, acompanhado pela implantação do Instituto Soroterápico Federal, em 1900, atualmente *campus* da Fundação Oswaldo Cruz (Fiocruz), que constitui um marco histórico importante desse processo (Fernandes, Costa, 2013; Rio de Janeiro, 2005).

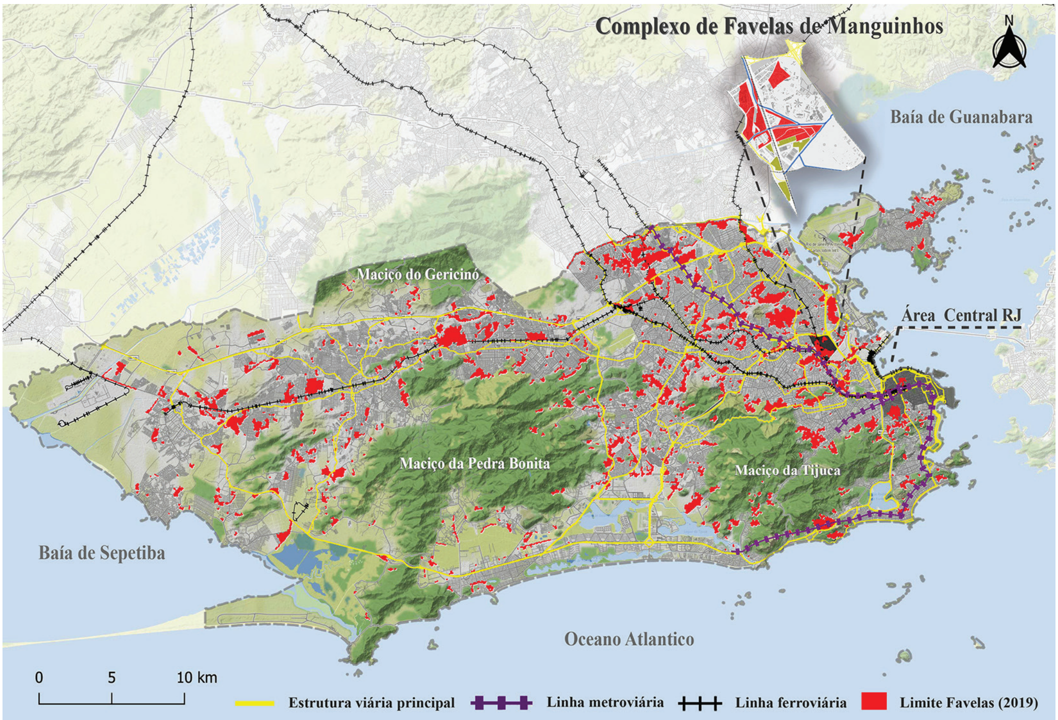

Figura 1: Mapa de localização do complexo de Manguinhos no município do Rio de Janeiro. Elaborado pelos autores sobre base cartográfica (2008) disponível no Google Earth (http://earth.google.com. Acesso em: 26 fev. 2023) e informações disponíveis no sistema de assentamentos de baixa renda Sabren/Data.rio (https://www.data.rio. Acesso em: 28 fev. 2023)

A ocupação no bairro de Manguinhos está no contexto do processo de expansão da ocupação urbana e de oferta de área disponível para a moradia popular, tendo sido objeto de estudo de vários trabalhos publicados que, em síntese, identificam os assentamentos precários que fazem parte do complexo de Manguinhos como um conjunto heterogêneo cujas especificidades internas refletem suas origens, perfis sociais, processos de produção do espaço habitado e um conjunto de aspectos políticos, econômicos e urbanísticos historicamente determinados (Fernandes, Costa, 2013; Rio de Janeiro, 2005; Soares et al., 2011; Trindade, 2012).

O início da ocupação de Manguinhos se dá em um contexto de crise habitacional que se verificou na cidade com a demolição de cortiços, desde o final do século XIX, e as reformas urbanas, no início do século XX, ações realizadas na área central, onde se concentravam as moradias de trabalhadores pobres e uma quantidade significativa de ex-escravizados.

A ocupação de áreas disponíveis nos subúrbios e na periferia da cidade, que dispunham de alguma estrutura de transporte, e nas áreas centrais, mesmo que não fossem adequadas à ocupação, impôs-se como necessidade. Esse processo de ocupação inicia uma nova tipologia de assentamento precário, a favela, denominação que muitas vezes tem servido para incluir, sob uma mesma representação social, territórios que apresentam características muito distintas (Barbosa, Silva, 2013, p.118-119).

Nas primeiras décadas do século XX, já há registro de fluxos migratórios expressivos para a região, mas o adensamento acelerado da área se verifica a partir das décadas de 1940 e 1950, quando se associam a implantação de empresas públicas e privadas, obras viárias, com destaque para a abertura da avenida Brasil, e o processo de aterramento das áreas marginais da principal via que atravessa o bairro e das áreas marginais dos rios que a cortam (Soares et al., 2011; Fernandes, Costa, 2013). O adensamento dessa região seguiu o processo de industrialização e expansão urbana da cidade, tendo recebido muitos assentamentos de famílias pobres removidas de áreas mais valorizadas da cidade. O intenso processo de industrialização, nas décadas de 1960 e 1970, acentuou o processo de ocupação populacional, principalmente por parte dos segmentos de baixa renda, que passaram a ocupar áreas cada vez mais desvalorizadas, com restrição à ocupação e que oferecem maior risco ambiental e, portanto, à saúde da população. Essas situações em Manguinhos são numerosas e diversas, considerando o conjunto de áreas alagáveis e próximas a aterros sanitários, às faixas marginais de rios e da linha férrea, às faixas não edificáveis da tubulação adutora de água da Companhia Estadual de Águas e Esgotos e das linhas de transmissão de energia elétrica de alta tensão.

O processo de urbanização e ocupação do território em Manguinhos reflete o processo de urbanização no Brasil que se deu, desde o início, sobre uma matriz marcada pela segregação social e a exclusão socioespacial. Como afirma Kowarick (1979, p.59), foi com a industrialização da segunda metade do século XX, com a migração rural-urbana da população, principalmente do Nordeste do país, em direção aos polos industriais do Sudeste, que se exacerbou um cenário trágico, em que se verifica a associação entre exploração econômica e espoliação urbana, esta última compreendida como "a somatória de extorsões que se opera por meio da inexistência ou precariedade dos serviços de consumo coletivos que agudizam ainda mais a dilapidação que opera nas esferas produtivas". A ausência de suporte público ao processo de crescimento se soma ao fato de que, tradicionalmente, as políticas urbanas são destinadas à implementação de melhorias na cidade formal privilegiada, enquanto as obras essenciais e urgentes nas periferias pobres são preteridas.

A partir das décadas de 1980 e, particularmente, de 1990, a região onde o bairro de Manguinhos está localizado passa a verificar o fechamento ou a relocalização de empresas, movimento que tenderia a se acentuar ao longo do tempo, seja por conta de processos de reestruturação produtiva, vantagens locacionais oferecidas por outras áreas ou agentes públicos, ou ainda pela crescente violência urbana observada nos constantes conflitos entre os grupos organizados em torno do tráfico de drogas e da criminalidade, e entre esses e as forças policiais (Fernandes, Costa, 2013; Soares et al., 2011; Rio de Janeiro, 2005). Ao longo destas últimas décadas, entretanto, e apesar da diminuição da oferta de empregos e da renda, Manguinhos permanece registrando um forte crescimento populacional por meio da ocupação de novas áreas, algumas dessas a partir da ocupação de vazios urbanos, edificados ou não, entre eles os que se tornaram disponíveis ao longo do processo de desindustrialização, e por meio do adensamento de áreas já ocupadas nos assentamentos. Permanecem também as iniciativas do poder público de ocupar áreas do bairro com o reassentamento de populações que viviam em áreas de risco localizadas nesse e em outros bairros. Atualmente, essa região concentra algumas das maiores densidades urbanas e permanece com alguns dos mais baixos índices de desenvolvimento humano e vulnerabilidade social da cidade, conforme pode ser verificado nos mapas disponíveis na plataforma interativa elaborada pelo Instituto de Pesquisa Econômica Aplicada que tem como base os dados censitários do Instituto Brasileiro de Geografia e Estatística de 2000 e 2010 (Ipea, 2015).

Entre as iniciativas públicas que buscavam transformar, em parte, esse quadro de acentuada vulnerabilização das populações residentes nos assentamentos precários de Manguinhos, cabe o registro de algumas intervenções pontuais, que davam particular atenção aos problemas de saneamento básico, como a atuação do programa Prosanear, à construção de conjuntos habitacionais e edificação de unidades de reassentamento para famílias removidas de áreas já impactadas ou suscetíveis a diversos tipos de risco, por exemplo, de enchentes. Merece destaque, porém, a intervenção urbanística realizada ao longo do período de 2008 a 2016, concentrada em 2009 e 2013, pelo Programa Nacional de Urbanização de Assentamentos Precários, um dos eixos de investimento do Programa de Aceleração do Crescimento (PAC), que apresentou forte impacto sobre a realidade dos moradores (Figura 2).

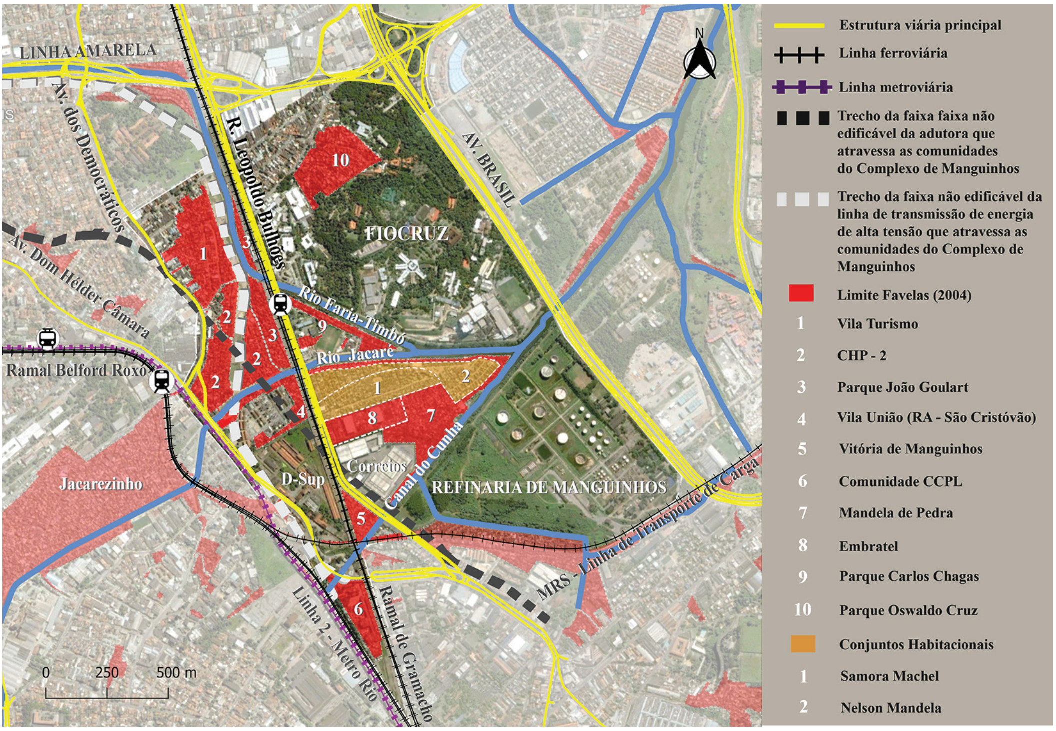

Figura 2: Mapa de localização das comunidades que foram objeto do PAC Manguinhos e sua inserção no contexto urbano. Elaborado pelos autores sobre base cartográfica (2008) disponível no Google Earth (http://earth.google.com/. Acesso em: 2 mar. 2023) e informações disponíveis no sistema de assentamentos de baixa renda Sabren/Data.rio (https://www.data.rio. Acesso em: 4 mar. 2023)

A escala do programa, também denominado PAC-UAP, não tem precedente quanto ao nível de recursos disponibilizados para a urbanização de favelas, por parte do governo federal, já que essa ação costumava ser, historicamente, da competência de estados e municípios, assim como não tem precedente o investimento em uma intervenção com esse escopo em Manguinhos. No período de 2007 a 2016, foram investidos recursos públicos da ordem de 686,2 milhões de reais que envolviam as três esferas de governo e tinham como operadores dos recursos o governo do estado e o município, cabendo ao estado o investimento prioritário em obras estruturais, referente à infraestrutura de transporte e saneamento, com ênfase na drenagem, equipamentos públicos e provisão habitacional. Ao município cabia a responsabilidade de realizar a urbanização das comunidades, a regularização fundiária e urbanística, equipamentos públicos e os reassentamentos de caráter local (Trindade, 2012).

O PAC Manguinhos representava uma oportunidade de garantir a transformação de alguns dos principais fatores do ambiente construído historicamente relacionados aos processos de determinação da saúde dessa população, entre eles os que impactam as condições concretas de vida, adquirem extrema materialidade no nível local (Ferreira, Buss, 2002) e permitem viabilizar o acesso ao direito à terra, à moradia digna e ao direito à cidade em suas condicionantes mais essenciais. As instruções normativas do programa previam também garantir as instâncias e os mecanismos de participação que poderiam oferecer à população a possibilidade concreta de disputar o futuro de seu bairro e, portanto, da cidade, assim como influenciar um processo de governança territorial democrática (Lima, 2017).

Como demonstra o conjunto de pesquisas de avaliação do PAC Manguinhos realizadas pela Fiocruz e outras instituições acadêmicas, junto a organizações da sociedade civil e moradores das comunidades, bem como registros de coletivos reunidos em instâncias de organização social, a esperança gerada nos moradores pelo início das obras do PAC Manguinhos, em um primeiro momento, deu lugar à frustração (Pivetta et al., fev. 2015). A implementação do programa voltou a refletir os problemas históricos que se verificam na execução de políticas públicas voltadas para as populações de favela. Entre eles estão a baixa qualidade e a provisoriedade como marca do planejamento e execução das obras, muitas mudanças nos projetos e problemas nos serviços realizados ao longo do processo, a falta de compromisso com a solução de problemas relevantes e a baixa importância oferecida aos moradores, diante dos impactos das decisões de projeto e obra sobre a sua vida (Pivetta et al., 2016). O abandono da área, após o ciclo das obras, sem as concluir adequadamente e sem considerar um programa de manutenção programada e de governança no território, acelerou a obsolescência e a percepção de degradação das obras, impactando a sua apropriação efetiva e afetiva, novamente reproduzindo o padrão de tratamento diferenciado oferecido às áreas nobres da cidade em detrimento das áreas populares.

A principal intervenção urbanística realizada, embora não representasse uma demanda prioritária da população, foi a elevação da linha férrea, responsável por um volume expressivo de recursos investidos. Já as intervenções que atuavam sobre o *deficit* e a inadequação habitacional, com atenção àquelas com foco no saneamento básico, embora fizessem parte do escopo e tenham sido objeto de intervenções, foram as que receberam as principais críticas de moradores e especialistas, ao lado daquelas que problematizaram a forma como se deu o processo participativo (Trindade, 2012; Lima, 2017; Pivetta et al., 2018).

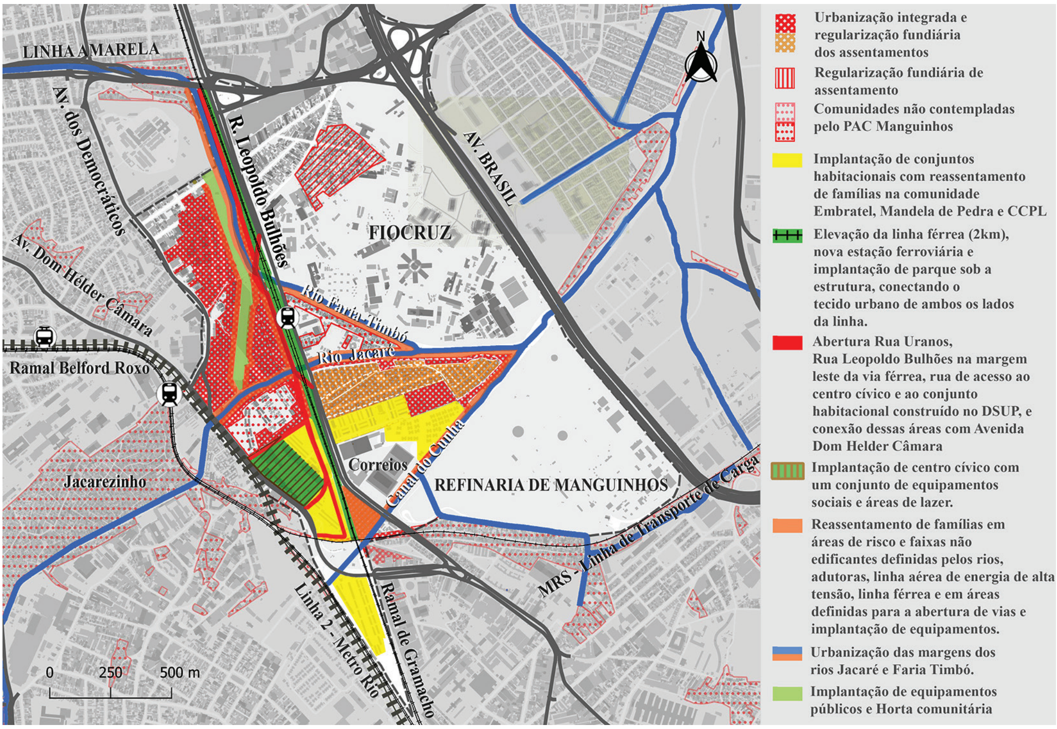

Figura 3: Mapa das principais intervenções do PAC Manguinhos. Elaborado pelos autores sobre base cartográfica constituída a partir de informações disponíveis no Google Earth (http://earth.google.com/. Acesso em: 8 mar. 2023), nas bases cartográficas de código aberto *terrain* no *website* Stamen (https://maps.stamen.com/terrain. Acesso em: 8 mar. 2023) e no Data.rio (https://www.data.rio. Acesso em: 9 mar. 2023), tendo como referência as informações do projeto de urbanização do PAC Manguinhos, considerando as intervenções efetivamente realizadas conforme análise documental e cartográfica

As enchentes em Manguinhos sempre foram uma preocupação central para a população diante das características geográficas, do modelo de urbanização adotado e da localização relativa do bairro, no estuário da bacia do canal do Cunha. São muitas áreas aterradas, de baixa declividade e cuja bacia hidrográfica recebe a contribuição pluviométrica de uma extensa área à montante, cuja superfície é predominantemente impermeabilizada, tendo como resultado um conjunto relevante de áreas ocupadas em Manguinhos com cotas suscetíveis a alagamento. Os problemas crônicos de enchentes representam sobretudo um risco à vida e à saúde, diante do alto impacto ambiental de efluentes e resíduos sólidos residenciais, incluindo entulho e lixo das obras, e das diversas atividades poluidoras, industriais e de serviços, lançados sem tratamento nos rios, bem como da possibilidade de contaminação dos sistemas de abastecimento de água e de proliferação de vetores, mas devem ser considerados também o impacto econômico nas famílias quando da perda de bens e os problemas gerados à conservação e eventual perda da própria moradia. Embora tenha sido definida uma estratégia para superar esse problema e estivesse claro, no plano de desenvolvimento urbanístico de Manguinhos (Rio de Janeiro, 2005), o entendimento de que as obras consideradas necessárias para a superação dos problemas de alagamentos seriam condicionantes para a urbanização, o que se verificou foram muitas enchentes ocorridas no processo de implementação e após a conclusão das obras, com forte impacto sobre a população (Pivetta et al., 2016; Soares, 2018; Alves, 6 jun. 2019; Temporal..., 30 abr. 2022). O problema das enchentes e o saneamento em Manguinhos permanece como um dos temas prioritários para seus moradores, como demonstrado na terceira Conferência Livre de Saúde em Manguinhos realizada nos dias 24 e 31 de julho de 2021.

A solução da drenagem urbana é condição para a implantação dos sistemas de esgotamento sanitário e abastecimento de água, principalmente considerando as características específicas da área. O comprometimento dos sistemas de esgotamento sanitário geralmente ocorre quando a implantação de um sistema separador absoluto, que distingue a captação e destinação das águas de chuva em relação às águas de esgoto, não é bem-sucedida, gerando problemas como o retorno do esgoto pelas tubulações domésticas e públicas, nesse caso, provocando a contaminação das superfícies de logradouros públicos e dos corpos d'água destinatários, principalmente rios e canais que banham a área. Com a permanência dos problemas das enchentes podemos supor o comprometimento progressivo e acelerado do sistema de esgotamento sanitário, situação acentuada por problemas de projeto e pela má qualidade das obras realizadas, como relatado por moradores (Soares, 2018; Pivetta et al., 2016; Alves, 6 jun. 2019).

O comprometimento dos sistemas de abastecimento de água ocorre, por sua vez, pela infiltração de água contaminada nas tubulações de água para consumo, que acontece principalmente quando há presença de água contaminada junto a essas tubulações, em áreas sujeitas a enchentes ou a vazamentos de esgoto. A qualidade do serviço de abastecimento de água também é impactada pela pressão insuficiente e intermitência na disponibilidade do recurso, conforme relatado por moradores (Pivetta et al., 2016). No escopo do programa no PAC Manguinhos não foi prevista a adequação das instalações hidrossanitárias das moradias, onde seria possível a implantação de tubulações distintas de águas pluviais e de esgotos sanitários e sua ligação adequada à rede pública (Soares, 2018), entre outras medidas de superação de inadequações no saneamento domiciliar das famílias, como a construção de banheiros e a previsão de instalação de caixas d'água nas unidades que não a possuem.

Considerando os resultados do Censo Domiciliar do Complexo de Manguinhos (Rio de Janeiro, 2010), realizado entre junho de 2008 e junho de 2009, podemos verificar alguns componentes da inadequação edilícia das residências, construções majoritariamente autoempreendidas e que apresentam um conjunto de problemas relatados pelos moradores como a inadequação ou insuficiência de espaços, de iluminação e ventilação natural em mais de 60% das moradias, presença de goteiras e umidade em paredes, chão ou fundação em mais de 45% e rachaduras em mais de 34% do universo pesquisado. Com as obras do PAC, a insegurança estrutural das habitações pode ter sido agravada, pois muitas moradias foram impactadas por trepidações geradas pelas obras, também provocando o rompimento de instalações elétricas, hidráulicas e de esgoto (Pivetta et al., 2016). Esses números revelam um impacto relevante das condições edilícias sobre a saúde da população e apontam para a importância de uma necessária ação pública para atuar sobre esse eixo componente das inadequações nas moradias que permaneceram após a intervenção realizada, considerando que nessas não foram realizadas melhorias habitacionais, mesmo constando no escopo do programa PAC-UAP.

A inadequação fundiária também representa um dos eixos de inadequação domiciliar, e a Região Metropolitana do Rio de Janeiro apresenta um dos maiores indicadores entre as regiões metropolitanas, equivalente a 12,15% dos domicílios duráveis, também concentrado na população de mais baixa renda. Além de um direito social, a regularização fundiária garante a segurança da posse, é estruturante para o acesso ao direito à cidade e o exercício da cidadania, bem como contribui com a gestão do território urbano. Na regularização fundiária plena, o processo de titulação, cujo instrumento estará apto para o registro imobiliário, consiste na regularização jurídica que deve ser articulada à regularização urbanística e ambiental.

No caso de Manguinhos, segundo informações da prefeitura, em 2018, o processo de regularização urbanística e fundiária abrangia áreas de favelas situadas nos complexos do Alemão, de Manguinhos e da Tijuca, além de outras áreas da cidade, como a Colônia Juliano Moreira (Rio de Janeiro, 2018). Segundo informações não documentadas recebidas da Coordenação de Regularização Fundiária da Secretaria Municipal de Habitação, entretanto, à exceção de alguns casos específicos cujos processos foram concluídos pelo governo do estado, os contratos foram encerrados sem a conclusão do processo, e, em 2022, estavam sendo realizados esforços para a formalização de um novo contrato visando retomar o processo de regularização fundiária nesses assentamentos.

Outro aspecto importante em relação às necessidades habitacionais se refere à demanda estimada e à provisão realizada de novas unidades habitacionais para o complexo de Manguinhos. Segundo o relatório que apresentou o balanço dos projetos do PAC em todo o Brasil, relativo a junho de 2014, o projeto apresentava um alto percentual de atendimento em relação à previsão consolidada de construção de 1.774 unidades habitacionais e a aquisição de 1.645 unidades para o reassentamento de famílias. Mesmo se considerarmos que as necessidades habitacionais de novas habitações seriam superiores a esse número, as famílias diretamente impactadas pelos processos de reassentamento habitacional representam um índice superior a 25% do total das, aproximadamente, 12 mil famílias beneficiadas pelo conjunto de ações desenvolvidas pelo programa em Manguinhos (Brasil, 2014). Isso denota a importância e a dimensão dos processos de decisão e de implementação desse item do escopo do projeto para a população.

As opções oferecidas para as famílias que eram removidas, em alternativa a uma nova unidade habitacional, demonstraram, por parte do poder público, não existir compromisso com o acesso à moradia digna, ao reforçar os processos informais e precários de ocupação. Nos processos de "indenização", na modalidade "compra assistida", onde o Estado acompanha e concretiza o processo de aquisição da nova casa, e no "auxílio aluguel", também identificado como "aluguel social", que consiste no valor pago às famílias que eram removidas e aguardavam o recebimento da nova unidade habitacional, os baixos valores oferecidos não permitiam que as famílias acessassem uma habitação em assentamentos formais e em melhor condição que a anterior, permanecendo essas famílias na precariedade, distantes de suas redes sociais e na incerteza sobre o futuro (Lima, 2018). A demora em receber as indenizações, na concretização da compra assistida, no recebimento das novas unidades habitacionais, perfazendo um longo prazo recebendo o auxílio moradia, e, ainda, a falta de definição sobre processos de remoção anunciados e não realizados acentuavam o sentimento de incerteza, abandono, frustração e a falta de confiança dos milhares de famílias que experimentaram esse processo de remoção (Pivetta et al., 2016). Cabe ainda o registro sobre a ausência do manejo adequado do entulho de casas demolidas, que permanecia no espaço de convívio das famílias do assentamento, contribuindo para a proliferação de vetores de doenças, num processo de clara violação de direitos (Lima, 2018).

No que se refere às unidades habitacionais construídas pelo PAC, apesar de os novos apartamentos terem significado uma real melhoria nas condições de moradia, sobretudo para aquelas famílias que moravam em unidades habitacionais com acentuada precariedade, uma questão inicial que merece nossa atenção é o fato de essas não corresponderem ao histórico padrão de ocupação dos assentamentos populares, como é a experiência de morar em Manguinhos. Esse padrão é tradicionalmente baseado em lotes unifamiliares, organizados em quadras ou vilas, que apresentam possibilidade de expansão do espaço construído com a construção sobre a laje da moradia, permitindo adaptação à dinâmica e às necessidades da família e cuja ocupação reflete a construção histórica de redes sociais. O padrão proposto das novas unidades habitacionais em blocos multifamiliares, com unidades padronizadas e gestão condominial, não se distancia das características dos programas de provisão habitacional que vêm sendo realizados há décadas, cabendo críticas semelhantes àquelas, já bastante consolidadas, referenciadas ao recente Programa Minha Casa Minha Vida (Amore, Shimbo, Rufino, 2015). Entre as críticas de especialistas e de moradores estão a baixa qualidade construtiva das unidades habitacionais, a existência de problemas relacionados ao projeto e à implantação dos prédios, a inundação de apartamentos do andar térreo, a infiltração de água de chuva pelas paredes, a informalidade no repasse dos apartamentos e na regulação e uso das áreas comuns, a permissividade na ocupação de espaços coletivos e logradouros públicos por novas habitações e comércio, bem como as dificuldades culturais, econômicas e administrativas impostas pela gestão condominial (Trindade, 2012; Lima, 2018).

O alto investimento realizado pelo programa PAC no território de Manguinhos se soma aos investimentos públicos que foram realizados no contexto da Copa do Mundo realizada em 2014 no Brasil e, mais especificamente, das Olimpíadas realizadas em 2016, que tinham na cidade a sua sede. Esses investimentos geraram um forte aquecimento do mercado imobiliário que se refletiu na valorização da terra, dos imóveis e no valor dos aluguéis, produzindo forte pressão sobre a renda familiar, com efeitos negativos sobre a segurança da posse e a imprevisibilidade sobre a permanência das famílias, bem como mudanças nas redes de sociabilidade. Para Abramo (2007), esse processo tem sido verificado nos assentamentos informais que são objeto de investimentos em projetos de urbanização de favelas, principalmente quando localizados próximo às áreas centrais, que apresentam maior oferta de emprego, equipamentos e serviços públicos, reforçando o processo de adensamento desses assentamentos. Nessas áreas, assim como em Manguinhos, o adensamento se verifica na ocupação de novas áreas, mesmo aquelas de onde famílias muito recentemente foram removidas e que se tornaram espaços públicos (Horta comunitária..., 3 out. 2022), assim como na verticalização das edificações. O adensamento também se verifica na subdivisão de imóveis e na expansão de unidades nos novos conjuntos habitacionais. As novas unidades habitacionais construídas informalmente apresentam, muitas vezes, área e padrão construtivo abaixo do que seria adequado e não conforme aos parâmetros da legislação urbanística e edilícia, sendo muitas delas destinadas ao mercado de aluguel. A dinâmica imobiliária informal também está associada aos desafios crescentes à gestão dos espaços públicos e ao controle sobre os espaços privados nos territórios controlados pela milícia ou pelo tráfico (Santos Junior et al., 2022).

Nos últimos anos, com o fim do ciclo olímpico e no contexto já mencionado de crise política, econômica e sanitária, esse processo de adensamento das comunidades, inserido em uma lógica informal de mercado e em suas lógicas endógenas, passa a ser pressionado pelo contexto de austeridade nos gastos públicos e cortes nos gastos sociais, em que se verifica a ausência de programas efetivos de enfrentamento do *deficit* e da inadequação habitacional. Esse contexto aponta para a obsolescência acelerada dos sistemas de infraestrutura e a degradação dos espaços urbanos e do estoque habitacional, sendo reforçada a percepção sobre a informalidade desses espaços. Assim, além das várias formas de violência perpetradas pelo aparato de segurança do Estado e pelos grupos armados que disputam o controle do território, os moradores de Manguinhos permanecem enfrentando as violências associadas ao não acesso ao direito à cidade e à moradia digna.

A partir de 2020, com a pandemia da covid-19, os processos de vulnerabilização e os riscos à população se agravaram, e os indicadores de inadequação domiciliar e de demanda por uma nova moradia explicitaram sua importância como mediadores de reprodução social das classes sociais no espaço social urbano, essenciais para a proteção da vida e da saúde da população. Se, na escala da família, a perda de empregos, a queda na renda e o crescimento acentuado da insegurança alimentar (Ahmed et al., jan. 2022) exigem um conjunto de medidas excepcionais e emergenciais de apoio às populações vulnerabilizadas para a superação das dificuldades de acesso às condições mínimas para o enfrentamento da pandemia, seja em casa ou fora dela, na escala da moradia e do *habitat* houve também uma convergência nos discursos sobre a urgência de constituição de medidas de curto e médio prazos de adequação na moradia e seu contexto urbano para a superação dessa histórica dívida social, como vocalizado pela Organização das Nações Unidas (ONU Mujeres, 2020). Nesse momento em que a pandemia da covid-19 parece próxima do fim, entretanto, a necessidade de medidas estruturais e transformadoras das iniquidades em saúde observadas na cidade e em Manguinhos enfrenta dificuldades em se constituir como prioridade e políticas efetivas.

## Considerações finais

A avaliação crítica do histórico urbanístico e social de Manguinhos aponta para a permanência dos processos de determinação social da saúde associados aos processos de produção e reprodução do espaço urbano e à perda de oportunidade de transformação efetiva da realidade vivenciada pela população em uma perspectiva promotora de saúde, mesmo diante dos vultosos investimentos recentes que foram realizados no território pelo PAC Manguinhos, que tinham como escopo uma forte atuação sobre as inadequações domiciliares e o *deficit* habitacional, e da trajetória histórica de resistência e luta por moradia da população de Manguinhos.

A crítica principal não se refere ao escopo do programa ou aos recursos que foram reservados para a intervenção, mas ao processo de implementação do programa. O fator essencial e que converge em todas as análises já realizadas sobre a implementação do PAC Manguinhos aponta para a não inclusão efetiva da população no processo participativo e no controle social das etapas desse processo. A questão principal, assim, não seria técnica, mas política. Esse fator influenciou negativamente todo o ciclo de planejamento e projeto, em que podemos citar desde o não aproveitamento de contribuições dos moradores ao diagnóstico da área e na definição e avaliação de alternativas projetuais nas diversas escalas, nas decisões de projeto em cada comunidade e para o bairro como um todo; a definição equivocada de prioridades, em desrespeito às demandas comunitárias históricas; a falta de informação e o desrespeito aos prazos a ser considerados nas obras, gerando tensões e conflitos desnecessários com moradores; a permissividade inaceitável sobre problemas técnicos observados nos projetos e na realização de obras; a ausência de procedimentos avaliativos ao longo do processo de implementação que pudessem aprimorar decisões projetuais e de obra; e o reforço de relações de poder existentes no território que representaram limites para a organização e a participação social pautada na cidadania e no processo emancipatório da população.

Entre outros fatores a considerar em uma perspectiva crítica sobre políticas públicas que lidem com a urbanização de assentamentos precários, o controle social e os processos participativos devem ser condição para a disputa política, seja em instâncias formais ou fora delas. Apontamos também a necessidade de uma perspectiva de atuação dessas políticas que garanta a durabilidade das ações e seus resultados, para além do momento da implementação do projeto. Uma atuação pública durável permite não somente a manutenção de investimentos realizados, mas a qualificação do ambiente construído e o aprimoramento da governança territorial, desde que submetidos à gestão democrática. Da mesma forma, deve-se buscar garantir a integração e a intersetorialidade nas ações públicas de planejamento e de intervenção no território, e as oportunidades existentes de uma ação articulada, com atenção especial ao setor saúde, considerando a estrutura estabelecida e a experiência consolidada da Estratégia de Saúde da Família, diante da coerência e das vantagens recíprocas que suas características podem apresentar na relação com uma estratégia de política urbana orientada por uma ação territorializada, participativa e promotora de saúde.

Podemos considerar que é possível adotar um programa de melhoramento integral de bairros saudáveis e sustentáveis, em uma perspectiva crítica de saúde urbana e centrada nos processos de determinação social da saúde, com o compromisso de atuar sobre as inadequações domiciliares e sobre o déficit habitacional, compreendendo a habitação como o espaço essencial e o veículo da construção e desenvolvimento da saúde da família. Não se trata somente de atuar sobre as necessidades habitacionais presentes e futuras da própria localidade, mas também sobre as necessidades habitacionais da cidade, compreendendo que essa atuação, compromissada com os marcos do direito à cidade, deve associar estratégias diversas e interescalares, invertendo a lógica de produção e reprodução do espaço urbano, atuando com centralidade sobre as desigualdades socioespaciais, distribuindo de forma justa e compensatória os ônus e bônus da dinâmica urbana, priorizando o orçamento e a atuação pública sobre as populações mais vulnerabilizadas em assentamentos precários. Esse caminho buscaria alterar qualitativamente as condições de enfrentamento dessa e de futuras emergências sanitárias e climáticas por essas populações, fortalecendo sua resiliência e sua capacidade de adaptação para condições potencialmente mais adversas a enfrentar no futuro.

## NOTAS

[1] O *deficit* habitacional deve ser compreendido como a necessidade de nova habitação estabelecida por meio de vários critérios, como a precariedade do domicílio, por ser rústico, improvisado ou apresentar dificuldades de acesso; os elevados custos com aluguel; e a coabitação mantida pela impossibilidade de acessar um novo domicílio (Fundação..., 2021a).

[2] Segundo a Fundação João Pinheiro (Fundação..., 2021b), os domicílios classificados como inadequados se referem aos domicílios duráveis urbanos, entendidos como aqueles que não compõem o *deficit* habitacional, portanto, não são considerados os domicílios improvisados ou rústicos e os cômodos. A inadequação é analisada segundo três critérios de inadequação: infraestrutura urbana, inadequação edilícia e inadequação fundiária.

[3] Conforme o Comitê dos Direitos Econômicos, Sociais e Culturais da ONU, de 1991, o conceito de moradia digna equivale ao de moradia adequada e aponta critérios que devem ser atendidos ao lado da disponibilidade de habitação, que compreendem segurança da posse; disponibilidade de serviços, materiais, instalações e infraestrutura; economicidade; habitabilidade; acessibilidade; localização e adequação cultural (Brasil, 2013, p.13).

[4] A Política Nacional de Habitação aborda tipologias típicas dos assentamentos brasileiros, e, no conceito de precariedade adotado, encontram-se quatro das categorias mais representativas do fenômeno, entre elas as favelas; os loteamentos irregulares e clandestinos de moradores de baixa renda; os conjuntos habitacionais produzidos pelo setor público, em situação de irregularidade ou de degradação; e os cortiços (Cardoso, 2008).

[5] A denominação Complexo de Manguinhos se refere não somente às favelas que estão presentes no bairro de Manguinhos, mas inclui os assentamentos precários considerados objeto de intervenção do PAC Manguinhos que não fazem parte de sua delimitação administrativa.
